# Exploring pathways to compulsory detention in psychiatric hospital and ways to prevent repeat detentions; Service user perspectives

**DOI:** 10.1371/journal.pmen.0000417

**Published:** 2025-09-22

**Authors:** Mary Birken, Ariana Kular, Patrick Nyikavaranda, Jordan Parkinson, Lizzie Mitchell, Kathleen Lindsay Fraser, Valerie Christina White, Janet Seale, Jackie Hardy, Cady Stone, Mark Keith Holden, Tyler Elliot, Zishi Li, Henrietta Mbeah-Bankas, Lisa Wood, Fiona Lobban, Brynmor Lloyd-Evans, Sonia Johnson

**Affiliations:** 1 Division of Psychiatry, Faculty of Brain Sciences, University College London, London, United Kingdom; 2 Department of Primary Care & Public Health, Brighton & Sussex Medical School, University of Sussex, Brighton, United Kingdom; 3 Lancashire and South Cumbria NHS Foundation Trust, Preston, United Kingdom; 4 FINCH Co-Production Group, Division of Psychiatry, Faculty of Brain Sciences, University College London, London, United Kingdom; 5 Education Reform, NHS England, London, United Kingdom; 6 North East London NHS Foundation Trust, London, United Kingdom; 7 Spectrum Centre for Mental Health Research, Faculty of Health & Medicine, Lancaster University, Lancaster, United Kingdom; 8 Camden and Islington NHS Foundation Trust, London, United Kingdom; PLOS: Public Library of Science, UNITED KINGDOM OF GREAT BRITAIN AND NORTHERN IRELAND

## Abstract

This study, co-produced by a team of academics, lived experience researchers and clinicians, explores the views and experiences of people who have been compulsorily detained in hospital under the Mental Health Act (1983) (MHA) in England, to understand how and why, from their perspective, compulsory detentions occur, and what might help prevent them. Semi-structured qualitative interviews were conducted with 20 people (55% male, 40% Black/Black British, 30% White British) who had been compulsory detained in hospital within the past 5 years. Lived experience researchers with relevant personal experience carried out interviews via telephone or videoconference and participated in analysis of data via a template approach. We derived three over-arching themes from interviews. The first theme “Individual factors increasing or reducing likelihood of being detained” encompassed factors related to people’s own lives and attitudes, including life stressors, not taking medication as prescribed, the risk individuals may pose to themselves or others, and their attitude to and management of their mental health. The second theme “Family and Social Network” reflects how attitudes and quality of support from family, friends and social network may contribute to compulsory detentions or help people to stay well. The third theme “Need for improvement in Service Responses” identified limitations of services that contribute to detention, including lack of collaborative care and choice, poor quality of professional support, and discriminatory attitudes from staff. Each theme also included potential approaches to addressing these limitations and reducing compulsory detentions. Findings suggest multiple interacting factors may lead to people being detained in hospital under the MHA, and that improvements to services, such as increasing collaborative care and service user-led family involvement, could prevent further detentions.

## Introduction

Compulsory detention in mental health wards is increasing in some higher income countries, including England [1–3]. Reducing compulsory detentions in hospital is a national and international priority as it is often a distressing and traumatising experience for service users and their carers, and may lead to prolonged and disrupted recovery and poor therapeutic alliances [4–5]. Compulsory detentions are also expensive, recently estimated as costing £18,315. equating to approximately €20,100. or $23,000. per detention, diverting resources from longer term preventive and recovery-approaches that may have better-established positive impacts [6].

While service users report some positive outcomes from compulsory detentions [4], experiences appear predominantly negative [4,7,8]. Compulsory detention by its nature infringes principles of informed consent, collaboration and joint decision making otherwise deemed essential in mental health care [9]. Mitigating harms, optimising experiences and outcomes and preventing further detentions where possible is thus a high priority.

In the UK, Section 2 or 3 are the most frequently used provisions in the Mental Health Act (1983) (MHA), allowing people to be detained in hospital for a specified period where it is deemed necessary for their own health or safety, or protection of others, while the individual is assessed, or when treatment cannot be given unless detained [10].

In England, there are large ethnic inequalities in risk of being detained under this legislation, with people from Black Caribbean, Black African, and Black British backgrounds having an estimated four times higher risk than White British individuals [11–12]. Qualitative research has found that Black people who have been detained often feel that the decision to detain them and coercive or traumatic experiences in hospital are linked to racism [13]. The UK government’s Independent Review of the Mental Health Act 1983 summarised complex factors associated with the detention of people from Black ethnic backgrounds, including structural disadvantages, lack of cultural awareness among mental health staff, and racism [6]. Reducing risk of detention is thus especially important for these minoritised ethnic groups as they are over-represented amongst those detained under the Mental Health Act.

Currently, research evidence on how to prevent detentions and repeat detentions remains very limited, with some evidence suggesting crisis planning-based interventions may reduce repeat detentions, but little other positive evidence to build on [14,15]. An enhanced understanding of risk factors for, and pathways to, compulsory detention has potential to inform interventions to reduce detentions. Quantitative studies yield some evidence on what is associated with greater risk of compulsory detention. Systematic reviews of risk factors for detentions among adults have identified a diagnosed psychotic disorder, previous episodes of involuntary hospitalisation, belonging to a minoritised ethnic group, especially Black British, Caribbean and African groups, male gender, unemployment, single marital status, not owning a home, and receiving state benefits as risk factors [11,16]. A recent narrative review [17] focused on quantitative evidence regarding contextual and societal factors that may be drivers of rates of compulsory admission, including limitations in services, such as lack of alternatives to admission, and societal factors such as austerity and high unemployment. Most of the literature included in reviews focuses on compulsory detention in general rather than repeat compulsory admission, but an investigation of repeat compulsory admission in the Netherlands found that previous history of mental health treatment or homelessness and poor self-care were among risk factors [18]. Awareness of risk factors is useful for development of preventive interventions, in that groups at high risk of detention can be focused on, but quantitative evidence so far yields only limited explanations of mechanisms underpinning these risk factors and pathways to compulsory admission [19,20].

Qualitative research has potential to contribute to understanding risk factors for and pathways to compulsory detention from the point of view of those detained, as well as families and clinicians. However, qualitative investigations of experiences of compulsory detention have tended to focus on experiences of being a detained inpatient [4] or on decision making at the point of admission [21] rather than on perceived reasons for and pathways to detention. In a Norwegian study using a grounded theory approach, participants reported that professionals tended to see their mental health difficulties in medical terms rather than in the context of their lives and social circumstances [22]. The consequent lack of focus on helping people address their social difficulties was seen as contributing to the crises resulting in detention [22]. Participants in this study also reported that doctors did not have enough time during consultations to properly explore patients’ circumstances.

Thus, only very limited evidence is currently available on patients’ accounts of pathways to compulsory admissions, and on their explanations for how such admissions come about and how they could be prevented. Our aim in this study was to provide novel evidence to address this gap, thus contributing to the development of approaches to reducing repeat compulsory hospitalisations. The present study is part of the first phase of an NIHR (National Institute of Health and Social Care research) funded study, the FINCH study (NIHR201739). Our overall aim in the FINCH study was to adapt an existing intervention based on crisis planning that showed promise in reducing compulsory detentions in a study in Switzerland [23], for use in the UK, and to test it in a feasibility trial. Our findings from the current study informed the adaptation of the intervention by a co-production group including service users, clinicians and researchers. We describe the intervention development process and the trial protocol in a separate paper [24]. By interviewing people who had been compulsorily detained in England in the past 5 years, we aimed to understand how and why, from their perspective, compulsory detention occurs, and to explore potential pathways to prevent compulsory detention. We also conducted a study exploring experiences of staff working in mental health services with people who have been involuntarily admitted to hospital under the Mental Health Act and is reported this in a separate paper [25].

## Methods

### Design

A co-produced qualitative study using semi-structured interviews was conducted. The research team held a range of relevant experience, and all contributed to design, delivery and writing up of the study. Ethical approval to conduct the study was received from UCL Research Ethics Committee (Ref: 15249/002).

### Research team

The design, conduct and analysis of the study was co-produced by Lived Experience Researchers (LERs) who are members of the FINCH Co-Production Group, and by other members of the FINCH study research team. All LERs had personal experience of using mental health services and of compulsory detention, and/or of supporting and caring for a relative or friend who had experienced compulsory detention. The FINCH Co-Production Group, consisting of LERs, carers, clinicians and researchers (some with multiple roles), was established at the start of the FINCH study, and met fortnightly through the first year of the FINCH study to plan the current study and to develop the FINCH intervention and methods for our feasibility trial. The LERs received training in conducting online interviews, analysis and obtaining verbal informed consent. A monthly lived experience reflective space provided LERs with emotional support and space to discuss the research process and its emotional impact. As study involvement lead, PN facilitated this, supported by senior members of the research team.

### Participants

Adults in England who had been detained under section 2 (mental health assessment) or section 3 (treatment for mental illness), of the Mental Health Act once or more in the last 5 years, were aged 18 years or over, and had capacity to consent, were eligible to take part in the study. The 5-year cut-off was intended to balance feasibility of recruiting a diverse sample with ability to recall events and relevance to current practice. We also felt that some participants may find it less distressing to talk about experiences of coercion when some time has passed. Purposive sampling was used to ensure diversity regarding participants’ ethnicity, gender, age, self-reported diagnosis, geographical location, and number of times detained. We reviewed our sample during recruitment and implemented targeted strategies to ensure diversity. These included approaching community organisations working with Black and minoritised ethnic communities and asking them to inform those using their services of the study and invite people to contact the researchers if they were interested in taking part.

### Recruitment

We contacted several community organisations, for example, Black Thrive, based in a borough in South London with high representation of people from Black ethnic groups, and IRIE MIND which is run by and for the African-Caribbean community in Hackney, London. We also contacted national charities that support people with mental health problems, such as several MIND centres in England, asking them to share the study poster with people accessing their organisation. We also asked the National Survivor User Network (NSUN), a network of individuals and user-led groups with lived experience of mental ill-health, distress, or trauma based in the UK, to disseminate the study adverts. We used X, formerly known as Twitter, to disseminate an invitation to the study through personal, study and institutional accounts, and asked organisations with Twitter accounts, Facebook and Instagram accounts to disseminate the study adverts. Potential participants contacted the research team by email. Researchers then checked eligibility, provided a participant information sheet, answered questions about the study, prior to booking interviews for those eligible and interested in taking part.

### Data collection

Before obtaining and recording informed verbal consent, capacity to consent was assessed, including by asking questions to ensure participants had fully understood the purpose and content of the interview.

The semi-structured interview topic guide was developed collaboratively by the Co-Production Group and the FINCH research team. We aimed to explore each participant’s most recent experience of being detained in hospital under section 2 or 3 of the Mental Health Act and of the support received following discharge, and to understand their views about what might have prevented them from being detained. The topic guide also asked participants’ views on the intervention being developed within the FINCH study, this further informed intervention development but is not reported in this paper. Interviews were conducted between September and December 2021. Please see S1 Appendix for the topic guide.

The interviews were conducted by five LERs with another FINCH researcher present to support recording and ensure the recording was securely stored in password protected university files. The interviews were conducted on Zoom or Teams videoconferencing platforms, and participants also had the option to phone in to Teams via a freephone number.

Interviewers were trained to be aware of the potentially sensitive content of the interview, and checked at intervals that participants were happy to continue and fully aware of the right to stop at any point. At the end of each interview, interviewers enquired whether the participant was comfortable with the interview, and offered a check in call a few days later. A list of services providing mental health support was provided to participants. Socio-demographic information was collected via a secure online survey on the Opinio survey software. All interviews were recorded and were transcribed verbatim. The duration of the interviews was between 30 minutes and one hour. All transcripts were then checked by the researchers and any identifying information was anonymised.

### Data analysis

The socio-demographic information collected online via Opinio was summarised by category and tabulated to describe the sample.

In keeping with a coproduction approach to the analysis of the interview transcripts, and to ensure interviews could be analysed by multiple analysts, we used template analysis [26], a form of thematic analysis [27]. This approach has been used successfully in health research [28], and also previously by some of the research team [29]. Template analysis involves the initial development of a coding template, based on a subset of transcripts, which is then applied to further transcripts and revised and refined as more data is analysed. This approach ensures a focus on collaboratively defining meanings and structure of themes during the process of analysis [26]. A coding template was used to organise the development of themes and final themes. The template was in the format of numbered versions of tables in word documents. The version number was changed after each round of analysis by the team of analysers and when themes changed following team discussions of themes. The twenty transcripts were analysed by a group of eleven researchers, eight of whom were Lived Experience Researchers. We didn’t formally check inter-rater reliability of coding but several transcripts were analysed by more than one person, which served to provide peer support to Lived Experience Researchers with less experience of analysis and support consistency among researchers in the coding process. Preliminary analysis of six transcripts was undertaken by six LERs to develop themes and subthemes as a basis for the first version of the analysis framework. A further eight transcripts were analysed by LERs and FINCH researchers, and an analysis meeting took place to revise the themes and subthemes in the analysis framework. The remaining transcripts were then analysed using the revised framework. A final analysis meeting was held to further review the analysis framework to merge similar themes or subthemes, or add new themes identified.

### Reflexivity

The research team comprised of Lived Experience Researchers, with experience of using mental health services and of compulsory detention, and/or of supporting and caring for a relative or friend who has experienced compulsory detention, and other researchers included clinical academics and non-clinical researchers from a range of backgrounds, including a psychiatrist, psychologist, social worker, and an occupational therapist. The research team members are from diverse backgrounds in terms of ethnicity, age, and gender. The plurality of perspectives within the team generated critical conversations about the nature and causes of repeat detention, with potential to challenge entrenched assumptions. LERs brought first-hand insights into the social, cultural, and racialised dimensions of mental health detention, including the postcode lottery of care, systemic inequalities, the impacts of experiences of racism, the challenging relationships of certain communities with the police, and social adversities as contributors to mental health crisis. Clinicians contributed an understanding of constraints when working in a stretched mental health system and of the pressures of risk management. These different understandings and the tensions between them shaped both the development of interview tools and discussions about the identification of themes and interpretation of findings. LERs led the interview process, so that their concerns may have especially shaped the manner of enquiry and the follow-up probes used at interviews.

## Results

### Participant characteristics

We recruited 20 people, of whom 11 (55%) were male and the most common age range was 25–29 years (35%). The most frequent groups were Black/Black British (40%), with White British (30%), and Mixed/multiple ethnic groups (15%) the next most represented ethnic groups. Half of the sample (50%) lived in London, with 60% living alone. Seventy percent of the sample had been compulsory detained under the MHA more than once, and psychosis was the most common mental health diagnosis (35%). Table 1 presents more details of the participants’ demographic characteristics. The demographic survey questions can be found in S3.

**Table 1:** Characteristics of Participants (N = 20).

Characteristics	Category	Number (%)
Age	18-24	2 (10%)
	25-29	7 (35%)
	30-34	0 (0%)
	35-39	5 (25%)
	40-44	0 (0%)
	45-49	1 (5%)
	50-54	2 (10%)
	55-59	1 (5%)
	60+	2 (10%)
Ethnicity	White British	6 (30%)
	White other	1 (5%)
	Mixed/multiple ethnic groups	3 (15%)
	Asian/Asian British	1 (5%)
	Black/Black British	8 (40%)
	Other ethnic group	0 (0%)
	Data not collected	1 (5%)
Gender	Female	8 (40%)
	Male	11 (55%)
	Prefer not to say	1 (5%)
Living Situation	Living Alone	12 (60%)
	Living with other adult(s) and dependent children	2 (10%)
	Living with other adult(s) (friends, housemates etc.), no dependent children	3 (15%)
	Living with dependent children and no other adults	1 (5%)
	Living with a partner, no dependent children	1 (5%)
	Data not collected	1 (5%)
Region of UK	London	10 (50%)
	North West	2 (10%)
	South East	2 (10%)
	West Midlands	2 (10%)
	Wales	1 (5%)
	Data not collected	3 (15%)
Mental Health Diagnosis	Psychosis	7 (35%)
	Bipolar	3 (15%)
	Depression	3 (15%)
	Multiple Disorders	4 (20%)
	Other	1 (5%)
	Not Known	2 (10%)
Number of times Compulsory admitted to hospital	1	6 (30%)
	2-5	8 (40%)
	6-10	3 (15%)
	10+	3 (15%)

## Findings

Three overarching themes were identified, and within them sub-themes that relate to factors found to increase the likelihood of being repeatedly detained in hospital under the Mental Health Act (MHA), and suggestions for preventing this. Overarching themes related to the individual, family and social network, and improving service responses. A summary of each theme is outlined in Table 2. Further quotes relating to each theme and subtheme can be found in S2.

**Table 2:** Overview of Themes and Sub-Themes.

Theme	Sub-Themes
**Individual Factors increasing or reducing likelihood of being detained**	Life Stressors and Other Events Attitudes to Own Mental Health & Self-Management Skills Not Taking Prescribed Medication Risk to Individual and Others
**Family and Social Network**	Attitude and Support from Family and Friends Wider Social Networks as a Protective Factor to Maintain Mental Health
**Need for improvement in Service Responses**	Collaborative Care and Choice Quality of Professional Support Discriminatory Attitudes

### Individual factors increasing or reducing likelihood of being detained

This theme encompasses factors relating to participants’ own behaviour and feelings, as perceived by participants, that contribute to them becoming unwell and being detained in hospital under the MHA. Participants also suggested individual strategies that might help prevent future detentions under the MHA, and ways of supporting these.

*Life Stressors and Other Events*

Participants reported that both recent and past events and current social stressors contributed to them being detained in hospital under the MHA. Some participants could see patterns to their detentions under the MHA over time, for example, the influence of recurring stressors were identified, or seasonal patterns:

“*Usually, I will become unwell when it’s sunny….I sort of become elated and a bit more manic, with sunny weather”* [P6]

Work-related stress, health concerns, illicit drug use, recent or past recurring traumatic experiences, bereavement, breakdown of relationships and an accumulation of problems with housing and family were reported as contributing factors:

“*I was depressed about a lot of things, I had a lot of challenges facing me then, I lost a very very close friend of mine, he’s more like a brother to me, I lost him in a gun violence incident, it was really a traumatic experience for me….I was just really losing it*” [P14]

*Attitudes to own Mental Health & Self-Management Skills*

Some participants spoke of having a lack of awareness regarding the deterioration of their mental health at the time and viewed an inability to use self-management skills as a contributing factor to eventual detention. For example, one participant stated that at the point of their mental health worsening:

*“I had no consciousness to decipher my actions. I didn’t decipher my actions at that particular point. I was just freestyling.”* [P12]*.*

Some participants found it difficult subsequently to make sense of what happened at the time when they were being detained, making it difficult to know what to do differently to avoid being detained in hospital again:

*“It is so traumatic being sectioned for anybody, and no one ever really talks about it after. It’s a really strange thing because you’re discharged and you’re back with your old team and it all just, sort of, disappears and you think, “Did I really just go through all that? What’s going on?”* [P4]

However, other participants described an increase in self-awareness regarding mental health following treatment in hospital:

*“I was never a kind of self-awareness person. I always saw things from a distance and I never really gained a lot of self-awareness. But after that* [creating a self-management plan] *I had to become a more self-aware person.”* [P12]*.*

Similarly others appeared to feel they could make an active choice to manage their mental health better post-discharge to avoid readmission:

*“Yes. I think... I just need to work on myself and bring myself back home. That was just it for me. And since my last sectioning, I think I’ve been doing pretty well. I’ve been able to manage all those mental stresses that I’ve been having and managing my family”* [P19]

Some participants wanted to take action to support themselves on discharge, to prevent further detentions under the MHA:

*“actually… I really don’t wish.. to get in a situation that could get me sectioned again, it was really an awful time and that’s why I am very, very intentional right now about managing my mental health.”* [P14]

*Not Taking Prescribed Medication*

Not taking prescribed medications was frequently identified as a factor leading to repeat detentions. Participants stopped taking medication for numerous reasons, including not liking the medication prescribed, for example due to feeling it was too strong; being unable to resist a strong urge to stop medication; being unsure what the medication was; feeling they did not need to take it; or voices telling them to stop taking the medication.

“*After a while, I get these voices that encourage me to stop taking them* (medication)*. And I wouldn’t tell anybody about it. I don’t even know why I stop the medication sometimes.”* [P16]

Having a regular weekly routine, particularly regarding medication, was identified as a strategy to prevent future detentions.

“*I think routine and a schedule to the week is always helpful so when it* (medication) *says evening, I take it in the early evening*” [P3]

*Risk to Individual and Others*

Participants reported engaging in behaviours, such as suicide attempts, which caused risk to themselves, then triggering a MHA assessment and detention.

Others reported behaving violently as their mental health declined, leading to detention.

“*So I just found myself really being this kind of violent, rowdy individual, fuming, and I had a lot of fury. It didn’t go well*.” [P10]

Illicit drug use and lack of self-care were other factors identified as leading to compulsory detention:

“*I was not, you know, in a very good shape at all. I wasn’t taking care of myself. At that point in time, I hardly was eating. I was actually very violent and I resorted to heavy drug consumption.”* [P19]

### Family and social network

This theme describes how an individual’s family and social network could impact positively or negatively on service users’ mental state and functioning, potentially either contributing to repeat detentions or helping to stay out of hospital.

*Attitude and Support from Family and Friends*

Some participants described having disconnected and poor relationships with family and friends, as contributing factors to repeat detentions. Some participants also described feelings of rejection, lacking social support, and feeling misunderstood and misrepresented by their family.

*“I think the issue is, once you’ve been labelled* (as having a mental illness)*, you’re disbelieved. So if you’ve got family around you who are sabotaging you and not being very helpful, and don’t have a label, they’re automatically believed.”* [P7]

This participant further stated they had a particularly negative relationship with their mother:

*“My mum has died now, but she was really, really abusive. But she was the one instigating it all. She was a little, old lady, and they believed what she had to say. So that’s quite difficult, I think. She’d be classed as the nearest relative.”* [P7]

In contrast, some participants described their family or friends as important in supporting them through their experience of mental ill-health.

“*Yeah, my dad’s like my rock and he really did help me through that experience* [process of appealing against section of Mental Health Act] [P13]

This included family or friends noticing changes in the participants’ behaviour and mental state, and encouraging them to seek help, intervening at the point of crisis, and supporting them through their Mental Health Act assessment by providing information about their mental health history to professionals:

*“I think they* (work friends) *made a difference because they were there to offer my history. They were there to provide my history to the medics, and even as I went through therapy, they gave me a realistic aspect of life”* [P15]

*Wider Social Networks as a Protective Factor to Maintain Mental Health*

Participants discussed how their wider social network had been beneficial in maintaining their mental health and thus preventing readmission. These included being in a positive employment role and engaging in external activities and community groups:

*“I was connected to friends because I was told* [by ward staff, when discussing discharge plans]*, “Do what you think can make you happy.” What could make me happy is playing football […] That really helped me because when I interacted with new friends and at least divert my energy to playing, it made me less distressed.”* [P11]

Some participants reported that workplace support and flexible working allowed them to maintain their employment:

*“I was encouraged by the fact that my employer decided not to fire me but, instead, decided to give me quite a relaxed timeline and timetable. My colleagues, also, were always there for me.”* [P14]

### Need for improvement in Service Responses

This theme encompassed problems in both inpatient and community services that may make compulsory detentions more likely to occur, and suggestions for improvements to address these. These challenges pertained to both inpatient detention and community-level care.

#### Collaborative care and choice.

Several participants emphasised the importance of being given choice in treatment, and felt this was lacking in all settings, making it less likely that treatment would be accepted voluntarily either in hospital or in the community. Regarding inpatient admissions, many said that medication was the primary treatment delivered rather than possible alternatives such as psychotherapy being offered, and that choices regarding which drugs would be used were not offered:

*“In hospital, they just gave me so many drugs, and I had no choice but to take them. They were forcibly given. I just complied, then, after that […] I only complied because if I didn’t, they’d have just kept injecting me with stuff. So I just ended up taking it.”* [P1]

There was also a reported lack of collaborative decision-making between service user and staff, for example in discussions at the point of detention:

*“there’s this me versus them. There was no sense of collaboration, no sense of, ‘We’re here to help you.’ It was, ‘We’re here to detain you,’ [...] That set the tone for the whole inpatient stay.”* [P1]

Some participants felt that they would have been less likely to be detained at their last admission if alternatives had been discussed:

*“It would have been helpful because I’m not someone who likes hospitals very well. The smell around hospital makes me very uncomfortable. I would have appreciated other options apart from being sectioned.”* [P16]

#### Quality of professional support.

Negative experiences of care from mental health services were described by several participants. Poor communication was a primary example, including staff not listening to the service user or their wider network.

*“my community team didn’t listen to the third sector organisation that was supporting me.”* [P9]

Some participants believed that if clinicians valued their voice, compulsory detention would probably have been avoided.

“*So their whole assessment and decision-making process really needs to be looked at. They ought to listen really to me and to the people who know me who I work with etc. because each time if they had done that there wouldn’t have been a hospitalisation, for the last three times.”* [P8]

Several participants also described staff as lacking interest in the individual service user; this included staff dismissing current care plans the service users wished to share with the team.

*“I had all my recovery plans and my crisis plans in my bag, and they did not even want to read it…But nobody has been interested in the last, I do not know, five years or so.”* [P9]

Crisis plans and having a structured care plan was seen as something that could have prevented previous compulsory detentions.

*“I think having a very structured comprehensive care plan in writing, accepting and going through it, and have, kind of, a crisis plan as well. Yes, I think, you know, I could have avoided many, many sections and many hospital interventions, because I could see the triggers, and I could see myself getting… I need to talk, and I need to, kind of, get a clear head, and avoid certain places or certain people.”* [P9]

Another example of poor-quality support was the lack of information provided by staff to service users regarding medication.

*“Well, there wasn’t any reasons for me to stop* [taking medication]*, I just felt I didn’t need them. I didn’t really have a clear understanding of what the medication was. I just believed the medication was for my mental health, maybe to help me get calmer”* [P19]

Participants also discussed a lack of continuity of relationships with staff.

*“It’s always different people, and then you just have to- even though they know the basics because they have your information, but you still end up having to repeat yourself again and again.”* [P20]

Many participants felt that being offered more post-discharge care, more accessible and available community support, alternatives to admission such as crisis houses, where they could feel safer and have more freedom, would have decreased the likelihood of them being compulsory detained on their last detention.

“*there is a really nice women’s crisis house around in XXX. They let you go out and they give you one-to-one in the morning*” [P2]

As well as in community settings, many participants felt that inpatient staff lacked interest in the service user at the point of discharge, including not providing a discharge or crisis plan, no information on medication withdrawal, and no acknowledgement of the individual’s external responsibilities.

*“They wouldn’t let me look at the bus number. I had barely any money. They didn’t help with that. They were really unhelpful. So I left the hospital with about £10 in my pocket, I had no phone, and I was withdrawing from all sorts: benzodiazepines, antipsychotics, mood stabilisers. So I was in quite a bad shape. But no, they didn’t give me any plan or help, or anything.”* [P7]

#### Discriminatory attitudes.

Discriminatory attitudes among staff were described by one participant. The participant described experiencing race discrimination and racial biases held by some clinicians.

*“I feel that it has a very strong element of racial discrimination there because the perception is, as a black woman at that, that I’m aggressive, that I’m difficult to deal with. And all of these things also have connotations with, “Black women act like that because they’ve got mental health difficulties.”* [P13]

## Discussion

### Main findings

Three over-arching themes were identified. These related to potential contributing factors to being detained in hospital at three levels: the service user, family and informal support network, and mental health services. Participants also shared suggestions to prevent further detentions.

The first theme “Individual factors increasing or reducing likelihood of being detained” reports external factors such as current and past life stressors and trauma, as well as internal factors such as individuals’ attitudes to and management of their mental health and decisions not to not take medication. Establishing routines including a medication regimen and self-management plans were reported as potentially preventing detention. The second theme “Family and Social Network” identified negative relationships with family as contributing factors to detention, and conversely having and keeping in contact with a supportive family, friends and wider network was seen as keeping people well and thus preventing detention. The third theme “Improving Service Responses” highlights issues with services such as a lack of collaborative care and choice, poor quality of professional support, and discriminatory attitudes by staff which contribute to detention and the necessity for these to be improved to lessen the prospect of compulsory detention. Thus, service user accounts suggest several types of contributing factors to compulsory detention at individual, social network and service levels, with a range of potential strategies for addressing them. Most of the strategies suggested by service users seemed to have some potential for implementation, including possible interventions to improve care planning and psychoeducation or alternatives to admission, although challenges such as lack of cohesive social networks maybe difficult for mental health services to influence. Whilst three over-arching themes reflecting different domains were identified by the coding team, there were multiple connections between the subthemes. For example, connections could be made between not taking medication as prescribed and to lack of continuity of support and collaborative decision-making, and lack of family support.

### Findings in the context of other studies

The focus of the current study on exploring service users’ views about what leads to compulsory admission is relatively novel: we are aware of little previous qualitative literature focusing on this particular question. An interview study in the UK exploring patients’ experiences of the assessment process identified lack of choice and of voice and involvement in decisions as central in these experiences [30]. Wormdahl et al. [22] carried out qualitative interviews and focus groups with patients and clinicians in Norway exploring pathways to involuntary admission: they identified a complex network of contributing factors, reflecting, as in our findings, both individual and service level difficulties, including living in deprived circumstances, discontinuing medication, and lack of responsiveness, collaboration and choice in mental health services. An important question is how far our findings reflect specific limitations of a national system: the above Norwegian study and our group’s reviews of international literature [4,31] suggest that at least some of the concerns we identified, such as lack of involvement in decision making and care planning and lack of understanding of the purpose of treatment, are relevant internationally. Lack of choice and coercion in varying forms has been reported in other literature to be especially characteristic of involuntary admission, and to be a feature of relationships with services for many people in many settings [32,33].

Our companion study exploring experiences of staff working in mental health services with people who have been involuntary admitted to hospital [25] allowed us to compare perspectives of clinicians and service users on contributing factors to compulsory admissions. Many of these overlapped, including at an individual level, medication non-adherence, racism, past trauma, and lack of supportive relationships, and at service level, poor continuity of care, limited treatment options, and discriminatory attitudes. Suggestions to reduce detentions were also aligned, with calls for more collaborative, person-centred care and better communication, and a wish for support networks to be stronger. Our findings also cohere with the available quantitative evidence base. For example, participants in our study reported a lack of choice at various stages in the pathway to admission and a lack of collaborative and supportive services. These findings mirror the associations found by Aluh et al [17] between compulsory admission, and poor-quality care and lack of admissions alternatives. Participants in our study also suggested that collaborative planning of care, for example for crises, has potential to reduce admissions. This is in line with the findings from synthesises of the literature on prevention of compulsory admissions that interventions based on crisis planning are thus far the only approach showing substantial evidence of effectiveness from trials [14,15].

A quantitative review of individual factors associated with compulsory admission [16] fits with some of the individual level factors identified by participants in our study including living in deprived circumstances, non-adherence to medication and behaviour becoming aggressive.

Participants from a range of ethnic groups across England took part in the study, and only one explicitly talked about experiencing racially discriminatory attitudes from staff which they perceived as contributing to them being detained under the MHA. This contrasted with findings from another qualitative study where people from a Black ethnic background in England were directly asked about racism and discrimination in the detention process, eliciting multiple reports of this [13]. Participants in our study did not report police involvement as a factor that contributed to their detention, whereas a review of quantitative studies of association found a strong association between police involvement in detention and involuntary care [16].

### Strengths and limitations

The current study had several strengths, including the novelty of the research question. There was considerable involvement from people with direct relevant personal experience who were involved as LERs in the planning, conducting and analysis of this study. Forty percent of the participants in this study were also from Black/Black British ethnic groups, whereas previous qualitative research [4] has been limited by the lack of participants from the most highly represented groups of those detained under the MHA. We didn’t directly ask about participants about police involvement in the path to detention, and therefore participants may not have considered this aspect in responding to questions. Our process for recruitment from a variety of non-health service sources may have missed some important perspectives. Additionally, limitations include having a heterogenous sample which limits the understanding of mechanisms and pathways for specific ethnic groups or other sub-groups of participants. Half of the sample were from London or one of four other UK regions, and 60% lived alone: this may have limited study representativeness. We did not collect detailed information from participants regarding the setting of admissions to hospital, or the duration of detentions; these may have provided rich detail to contextualise findings. Participants self-reported their diagnosis using broad response categories. This limited the detail we are able to report about participants’ specific diagnosis. We didn’t explicitly ask whether participants had experienced any other coercive interventions during the detentions, which may have impacted their experience, for example restraint, seclusion, forced medication. During the study interviews, many participants had difficulties recalling what happened during the MHA assessment process, and reflected relatively little on the discussions that took place at this time or what options were discussed during the assessment. Interviewing participants immediately after discharge may help support participants to recall the MHA assessment experience whereas participants in our sample had been detained up to 5 years ago. Furthermore, explicit questions about the cultural appropriateness of care or experiences of discrimination were not included in the interview topic guide as our approach was to ask very broad open questions: thus we may well not have elicited all that respondents could have said about this. The use of multiple interviewers and a large analysis team with a wide range of experiences and characteristics may have enhanced the validity of themes, identified from multiple perspectives, but may also have increased variability in how interviews and analytic coding were conducted.

### Further research

A number of potential strategies to reduce detention are supported both by our study and other relevant qualitative literature, including better support in the community, more choice and involvement in decisions and more effective care planning and availability of alternatives in a crisis. There is still very little evidence evaluating the effectiveness of interventions developed to reduce detention, or on the impact of different community support arrangements on detentions [14,17]. Thus, both trials of novel interventions to prevent detention among people at high risk, for example based on crisis planning and/or shared decision making, and naturalistic evaluations of the relationship between community and crisis care arrangements and detentions, are potentially of value. Although we ensured we had a diverse sample, including groups at high risk of compulsory detention, we did not specifically explore relationships between experiences of racism and discrimination or cultural identity and risk of detention; a more in-depth approach is likely to be beneficial.

#### Clinical and policy implications.

Participants’ views of what might contribute to and prevent compulsory detention were often practical in nature and fitted well with the published evidence, where available, supporting several potential pathways to preventing detentions and repeat detentions. At the individual level, service users’ views supported the potential value of supported self-management, and of interventions to alleviate problematic social circumstances, such as interventions to alleviate social isolation, or housing or employment difficulties [34,35], and the importance of clinicians taking into account advanced choices, also supported by national policy in England [36].

Some findings suggested that family involvement has potential value in preventing detention where the service user chooses to involve them, or at least that there are currently some potentially avoidable negative effects of family involvement.

Interventions that aim to involve family and friends in care such as Open Dialogue, may support patient-led involvement of families, although evidence is not definitive currently [37]. One of the measures in the Mental Health Bill in England and Wales [36], introduced to reform compulsory detention principles and processes, is that a patient can decide who should be consulted when treatment decisions are being made, rather than the current system where the law stipulates who should be regarded as “nearest relative” and thus involved in consultations. A further measure in the new legislation is more detailed risk assessment with a higher threshold for compulsory detention, requiring a substantial likelihood of significant harm, and a statutory obligation for detained service users to have a detailed and collaborative care and treatment plan [36]. Our findings are in keeping with these plans, currently (June 2025) at a late stage in becoming law in England and Wales.

At service level, the value of talking to service users about what is likely to be helpful is reflected in clear suggestions that they made for improvements at a several levels. Suggestions included implementation of crisis plans, supported self-management, and collaborative decision, all interventions with substantial research evidence of effectiveness [14,25,35]. Our findings also suggest that interventions to improve alliance with staff, service user voice and collaborative decision making at all stages are potentially valuable in preventing detentions [38]. Participants highlighted a lack of voice or collaboration in the assessment process, and this links with findings in a previous study [34] and highlights the need for initiatives to improve the MHA process. It also appears important that mental health staff ensure effective management of endings in mental health care wherever feasible, ensure continuity of care, and engage in collaborative decision-making. Discharge following a compulsory admission emerges as a crucial timepoint: formulation of clear care plans, education on medication and self-management, patient-led engagement with families and friends have potential to prevent further detentions. In the community, support with social difficulties and focusing resources on long term, high quality and individualised community care involving shared decision making also appears a potential strategy for reducing compulsory admission. When compulsory admission or readmission is considered, our participants’ findings suggest that the provision of community crisis alternatives is also helpful, although research evidence has not thus far demonstrated that such alternatives reduce compulsory as opposed to voluntary admissions [39,40]. A single participant referred to discrimination, but many participants felt disempowered and lacking a voice in the care they received an experience which could have been linked to racial inequalities, even though participants did not explicitly make this connection [41].

## Conclusion

This study has identified complex interacting factors that may contribute to people being detained in hospital under the MHA, and that strategies for improved services, including more collaborative care in addition to service user-led family involvement may prevent further detentions. Increasing collaborative care, self-management and family and wider support are likely to reduce further detentions. Interventions specifically aiming to reduce further detentions and focusing on these aspects of care need to be developed and tested.

## Supporting information

S1 Appendix Phase one interview topic guide service users. (DOCX)

S2 Appendix Service user paper supplementary quotes. (DOCX)

S3 Appendix Service user demographics survey questions. (DOCX)

## References

[1] Hatfield B, Antcliff V. Detention under the Mental Health Act: balancing rights, risks and needs for services. Journal of Social Welfare and Family Law. 2001;23(2):135–53. doi: 10.1080/01418030122309

[2] NHS Digital. Mental Health Act Statistics, Annual Figures, 2021-22. 2022a. cited 26 January 2023. https://digital.nhs.uk/data-and-information/publications/statistical/mental-health-act-statistics-annual-figures/2021-22-annual-figures

[3] Sheridan Rains L, Zenina T, Dias MC, Jones R, Jeffreys S, Branthonne-Foster S, et al. Variations in patterns of involuntary hospitalisation and in legal frameworks: an international comparative study. Lancet Psychiatry. 2019;6(5):403–17. doi: 10.1016/S2215-0366(19)30090-2 30954479 PMC6475657

[4] Akther SF, Molyneaux E, Stuart R, Johnson S, Simpson A, Oram S. Patients’ experiences of assessment and detention under mental health legislation: systematic review and qualitative meta-synthesis. BJPsych Open. 2019;5(3):e37. doi: 10.1192/bjo.2019.19 31530313 PMC6520528

[5] Stuart R, Akther SF, Machin K, Persaud K, Simpson A, Johnson S, et al. Carers’ experiences of involuntary admission under mental health legislation: systematic review and qualitative meta-synthesis. BJPsych Open. 2020;6(2):e19. doi: 10.1192/bjo.2019.101 32043435 PMC7176830

[6] Department of Health and Social Care. Modernising the Mental Health Act – final report from the Independent Review. London: DHSC. 2018.

[7] Aluh DO, Ayilara O, Onu JU, Grigaitė U, Pedrosa B, Santos-Dias M, et al. Experiences and perceptions of coercive practices in mental health care among service users in Nigeria: a qualitative study. Int J Ment Health Syst. 2022;16(1):54. doi: 10.1186/s13033-022-00565-4 36424651 PMC9694572

[8] Shozi Z, Saloojee S, Mashaphu S. Experiences of coercion amongst involuntary mental health care users in KwaZulu-Natal, South Africa. Front Psychiatry. 2023;14:1113821. doi: 10.3389/fpsyt.2023.1113821 36960456 PMC10027751

[9] National Institute of Clinical Excellence. Service user experience in adult mental health services. 2019. Cited 28 July 2023. https://www.nice.org.uk/guidance/qs14

[10] NHS. Mental Health Act. Accessed 12 Oct 2023. 2022. https://www.nhs.uk/mental-health/social-care-and-your-rights/mental-health-and-the-law/mental-health-act/

[11] Barnett P, Mackay E, Matthews H, Gate R, Greenwood H, Ariyo K, et al. Ethnic variations in compulsory detention under the Mental Health Act: a systematic review and meta-analysis of international data. Lancet Psychiatry. 2019;6(4):305–17. doi: 10.1016/S2215-0366(19)30027-6 30846354 PMC6494977

[12] NHS Digital. Detentions under the Mental Health Act. 2022b. cited 19 May 2023. https://www.ethnicity-facts-figures.service.gov.uk/health/mental-health/detentions-under-the-mental-health-act/latest#by-ethnicity-5-ethnic-groups

[13] Solanki J, Wood L, McPherson S. Experiences of adults from a Black ethnic background detained as inpatients under the Mental Health Act (1983). Psychiatr Rehabil J. 2023;46(1):14–20. doi: 10.1037/prj0000537 36809012

[14] Bone JK, McCloud T, Scott HR, Machin K, Markham S, Persaud K, et al. Psychosocial Interventions to Reduce Compulsory Psychiatric Admissions: A Rapid Evidence Synthesis. EClinicalMedicine. 2019;10:58–67. doi: 10.1016/j.eclinm.2019.03.017 31193820 PMC6543173

[15] Molyneaux E, Turner A, Candy B, Landau S, Johnson S, Lloyd-Evans B. Crisis-planning interventions for people with psychotic illness or bipolar disorder: systematic review and meta-analyses. BJPsych Open. 2019;5(4):e53. doi: 10.1192/bjo.2019.28 31530302 PMC6582216

[16] Walker S, Mackay E, Barnett P, Sheridan Rains L, Leverton M, Dalton-Locke C, et al. Clinical and social factors associated with increased risk for involuntary psychiatric hospitalisation: a systematic review, meta-analysis, and narrative synthesis. Lancet Psychiatry. 2019;6(12):1039–53. doi: 10.1016/S2215-0366(19)30406-7 31777340 PMC7029280

[17] Aluh DO, Aigbogun O, Ukoha-Kalu BO, Silva M, Grigaitė U, Pedrosa B, et al. Beyond Patient Characteristics: A Narrative Review of Contextual Factors Influencing Involuntary Admissions in Mental Health Care. Healthcare (Basel). 2023;11(14):1986. doi: 10.3390/healthcare11141986 37510426 PMC10379438

[18] de Jong MH, Wierdsma AI, Zoeteman J, van Boeijen CA, Van Gool AR, Mulder CL. Risk factors for repeated emergency compulsory psychiatric admissions. BJPsych Open. 2020;7(1):e19. doi: 10.1192/bjo.2020.153 33349278 PMC7791558

[19] Sheridan Rains L, Weich S, Maddock C, Smith S, Keown P, Crepaz-Keay D, et al. Understanding increasing rates of psychiatric hospital detentions in England: development and preliminary testing of an explanatory model. BJPsych Open. 2020;6(5):e88. doi: 10.1192/bjo.2020.64 32792034 PMC7453796

[20] Freitas DF, Walker S, Nyikavaranda P, Downs J, Patel R, Khondoker M, et al. Ethnic inequalities in involuntary admission under the Mental Health Act: an exploration of mediation effects of clinical care prior to the first admission. Br J Psychiatry. 2023;222(1):27–36. doi: 10.1192/bjp.2022.141 36281471 PMC10250681

[21] Sugiura K, Pertega E, Holmberg C. Experiences of involuntary psychiatric admission decision-making: a systematic review and meta-synthesis of the perspectives of service users, informal carers, and professionals. Int J Law Psychiatry. 2020;73:101645. doi: 10.1016/j.ijlp.2020.101645 33246221

[22] Wormdahl I, Husum TL, Kjus SHH, Rugkåsa J, Hatling T, Rise MB. Between No Help and Coercion: Toward Referral to Involuntary Psychiatric Admission. A Qualitative Interview Study of Stakeholders’ Perspectives. Front Psychiatry. 2021;12:708175. doi: 10.3389/fpsyt.2021.708175 34484000 PMC8415795

[23] Lay B, Kawohl W, Rössler W. Outcomes of a psycho-education and monitoring programme to prevent compulsory admission to psychiatric inpatient care: a randomised controlled trial. Psychol Med. 2018;48(5):849–60. doi: 10.1017/S0033291717002239 28805175

[24] Johnson S, Birken M, Nyikavaranda P, Kular A, Gafoor R, Parkinson J, et al. A crisis planning and monitoring intervention to reduce compulsory hospital readmissions (FINCH study): protocol for a randomised controlled feasibility study. Pilot Feasibility Stud. 2024;10(1):35. doi: 10.1186/s40814-024-01453-z 38378694 PMC10877855

[25] Kular A, Birken M, Wood L, Parkinson J, Bacarese-Hamilton T, Blakley L, et al. Exploring pathways to compulsory detention and ways to prevent repeat compulsory detentions in England; clinician perspectives. PLOS Ment Health. 2025;2(6):e0000314. doi: 10.1371/journal.pmen.0000314 PMC12798629 41661881

[26] King N, Brooks JM. Template Analysis for Business and Management Students. London: Sage; 2017.

[27] Braun V, Clarke V. Reflecting on reflexive thematic analysis. Qualitative Research in Sport, Exercise and Health. 2019;11(4):589–97. doi: 10.1080/2159676x.2019.1628806

[28] Brooks J, McCluskey S, Turley E, King N. The Utility of Template Analysis in Qualitative Psychology Research. Qual Res Psychol. 2015;12(2):202–22. doi: 10.1080/14780887.2014.955224 27499705 PMC4960514

[29] Birken M, Chipp B, Shah P, Olive RR, Nyikavaranda P, Hardy J, et al. Exploring the experiences of loneliness in adults with mental health problems: A participatory qualitative interview study. PLoS One. 2023;18(3):e0280946. doi: 10.1371/journal.pone.0280946 36881570 PMC9990944

[30] Blakley L, Asher C, Etherington A, Maher J, Wadey E, Walsh V, et al. “Waiting for the verdict”: the experience of being assessed under the Mental Health Act. J Ment Health. 2022;31(2):212–9. doi: 10.1080/09638237.2021.1922624 34006171

[31] Bartl G, Stuart R, Ahmed N, Saunders K, Loizou S, Brady G, et al. A qualitative meta-synthesis of service users’ and carers’ experiences of assessment and involuntary hospital admissions under mental health legislations: a five-year update. BMC Psychiatry. 2024;24(1):476. doi: 10.1186/s12888-024-05914-w 38937705 PMC11209989

[32] Hoge SK, Lidz CW, Eisenberg M, Gardner W, Monahan J, Mulvey E, et al. Perceptions of coercion in the admission of voluntary and involuntary psychiatric patients. Int J Law Psychiatry. 1997;20(2):167–81. doi: 10.1016/s0160-2527(97)00001-0 9178060

[33] Szmukler G, Appelbaum PS. Treatment pressures, leverage, coercion, and compulsion in mental health care. Journal of Mental Health. 2008;17(3):233–44. doi: 10.1080/09638230802052203

[34] Barnett P, Steare T, Dedat Z, Pilling S, McCrone P, Knapp M, et al. Interventions to improve social circumstances of people with mental health conditions: a rapid evidence synthesis. BMC Psychiatry. 2022;22(1):302. doi: 10.1186/s12888-022-03864-9 35484521 PMC9047264

[35] Lean M, Fornells-Ambrojo M, Milton A, Lloyd-Evans B, Harrison-Stewart B, Yesufu-Udechuku A, et al. Self-management interventions for people with severe mental illness: systematic review and meta-analysis. Br J Psychiatry. 2019;214(5):260–8. doi: 10.1192/bjp.2019.54 30898177 PMC6499726

[36] Department of Health and Social Care and Ministry of Justice. Draft Mental Health Bill. 2022. Retrieved 15 Nov 2023 https://www.gov.uk/government/publications/draft-mental-health-bill-2022

[37] Pilling S, Clarke K, Parker G, James K, Landau S, Weaver T, et al. Open Dialogue compared to treatment as usual for adults experiencing a mental health crisis: Protocol for the ODDESSI multi-site cluster randomised controlled trial. Contemp Clin Trials. 2022;113:106664. doi: 10.1016/j.cct.2021.106664 34958932 PMC8844585

[38] Bowers L, James K, Quirk A, Simpson A, Sugar Stewart D, et al. Reducing conflict and containment rates on acute psychiatric wards: The Safewards cluster randomised controlled trial. Int J Nurs Stud. 2015;52(9):1412–22. doi: 10.1016/j.ijnurstu.2015.05.001 26166187 PMC4518134

[39] Johnson S, Dalton-Locke C, Baker J, Hanlon C, Salisbury TT, Fossey M, et al. Acute psychiatric care: approaches to increasing the range of services and improving access and quality of care. World Psychiatry. 2022;21(2):220–36. doi: 10.1002/wps.20962 35524608 PMC9077627

[40] Dalton-Locke C, Johnson S, Harju-Seppänen J, Lyons N, Sheridan Rains L, Stuart R, et al. Emerging models and trends in mental health crisis care in England: a national investigation of crisis care systems. BMC Health Serv Res. 2021;21(1):1174. doi: 10.1186/s12913-021-07181-x 34711222 PMC8553397

[41] Smith SM, Kheri A, Ariyo K, Gilbert S, Salla A, Lingiah T, et al. The Patient and Carer Race Equality Framework: a model to reduce mental health inequity in England and Wales. Front Psychiatry. 2023;14:1053502. doi: 10.3389/fpsyt.2023.1053502 37215650 PMC10196047

