# Supplementary material for: Exploring pathways to compulsory detention in psychiatric hospital and ways to prevent repeat detentions; Service user perspectives
Source: PLOS Ment Health. 2025 Sep 22;2(9):e0000417. doi: 10.1371/journal.pmen.0000417 (PMC12798403; doi:10.1371/journal.pmen.0000417)
Supplement: S1 Appendix — (DOCX) [file pmen.0000417.s001.docx]

**Study: Exploring pathways to detention in psychiatric hospital and ways to prevent repeat detentions: a qualitative study**

**Interview Topic Guide for online semi-structured interviews with People with experience of being involuntarily admitted under the Mental Health Act**

**Introduction:**

**We are a research team, including mental health staff and people who have personal experience of using mental health services, and people who have caring experience. We are carrying out a study in which we are trying to develop a new type of support to help people who have been “sectioned” reduce their likelihood of being “sectioned” again in the future.**

**To help us develop this new type of support, we are trying to find out more about people’s experience of what led to them getting “sectioned”, and what you think could be helpful in stopping this from happening again.**

**We also want to ask you about our ideas for the new type of support and get your thoughts on how helpful and appealing it sounds, so we can try to make it as relevant and useful as possible.**

**We are aware that some of the questions are about difficult times in your life and could be upsetting – please only share what you feel comfortable talking about. We can take breaks during the interview so please let me know if you’d like to stop at any time.**

1. **When was the last time you were “sectioned in hospital”?**

**Can you please describe what happened in the run-up to being sectioned?**

*Prompt for: whether they think any stresses in their lives or any recent events that were out of the ordinary, had treatment and contact with services changed in any way, do they think their mental health was deteriorating? If they thought their mental health was deteriorating, did they know what to do? Also prompt for what happened on the day of the Mental Health Act assessment and on the days leading up to it, and what discussion they remember about options at this assessment or just before.*

1. **Do you think there is anything that could have made getting sectioned (again) less likely?**

*Prompt for: anything staff could have done, whether it would have helped to be in contact with different services, anything the person could have done themselves, anything family and friends could have done. Also, what kind of follow-up did they have after the last admission, and how was this? Did they drop out of care, and if so, what was done and could it have been different? Did they stop taking meds – if so, why did they stop taking meds, did they understand why it was recommended they take them? Were they having problems with the meds, and did they know how to get them reviewed? what was done after they stopped taking meds, and could it have been different? Ask about their care plan and risk management plan and whether they led the discussion on this and who was in charge of managing this in the community e.g. care co-ordinator etc.*

1. **Looking back to previous times you have been sectioned, did that follow a similar pattern? If not, what was different on past occasions?**

*Probe for any other risk factors, things that could be changed, different patterns of ending up back on section. Discuss some of the above without being leading.*

1. **When you last left hospital, did you have any kind of plan for staying well?**

**Were you given any guidance about keeping an eye on your mental health?**

**Did you know what to do if things got worse with your mental health?**

*Prompt for any kind of crisis plan, relapse prevention plan, risk management plan, community support,* *self-management plan, how they understand it and what they feel is being achieved. (Good to find out if a care, crisis plan, relapse plan was monitored or just produced without any follow up.)*
