# Supplementary figures and images for: Exploring pathways to compulsory detention in psychiatric hospital and ways to prevent repeat detentions; Service user perspectives
Source: PLOS Ment Health. 2025 Sep 22;2(9):e0000417. doi: 10.1371/journal.pmen.0000417 (PMC12798403; doi:10.1371/journal.pmen.0000417)

**FINCH Service User Demographics Survey Questions**


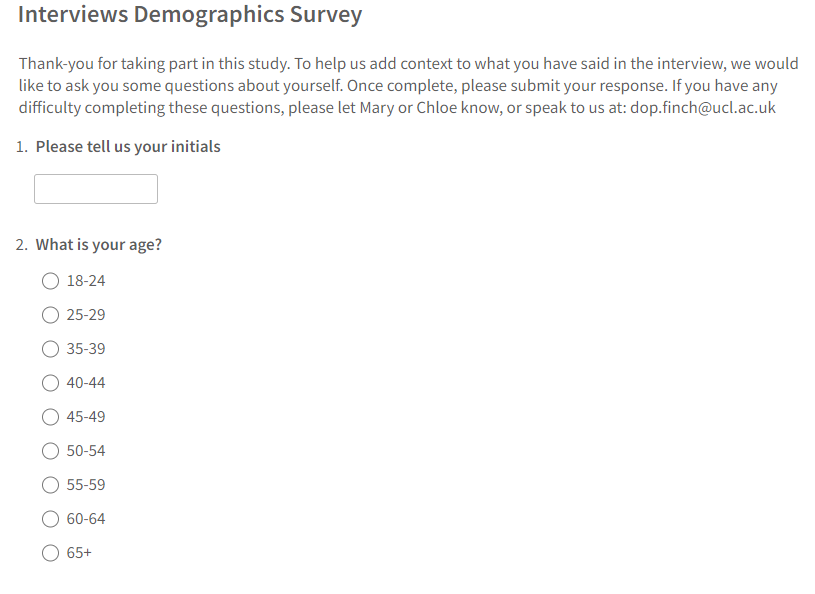


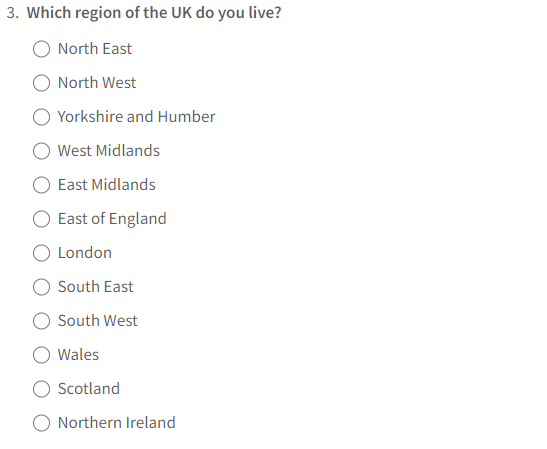


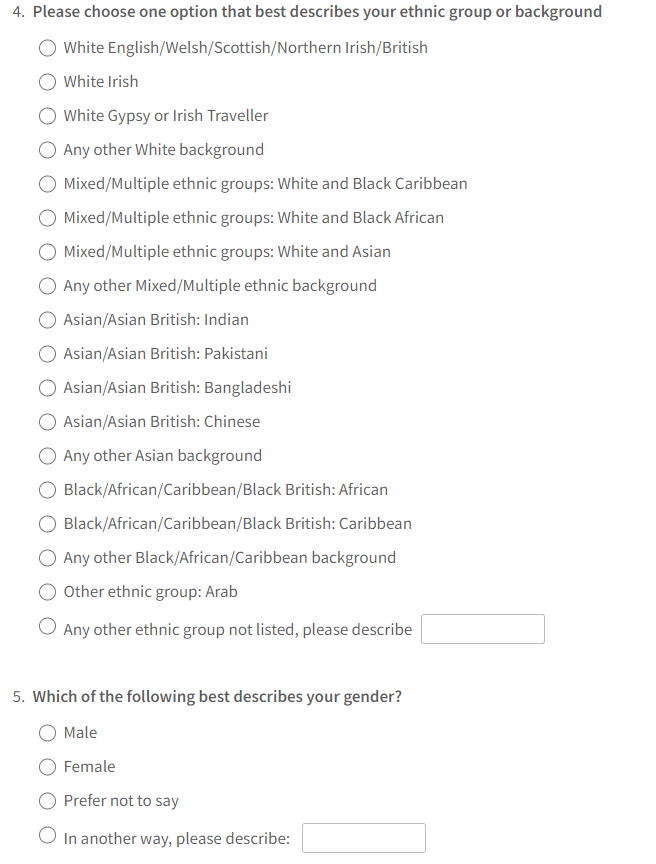


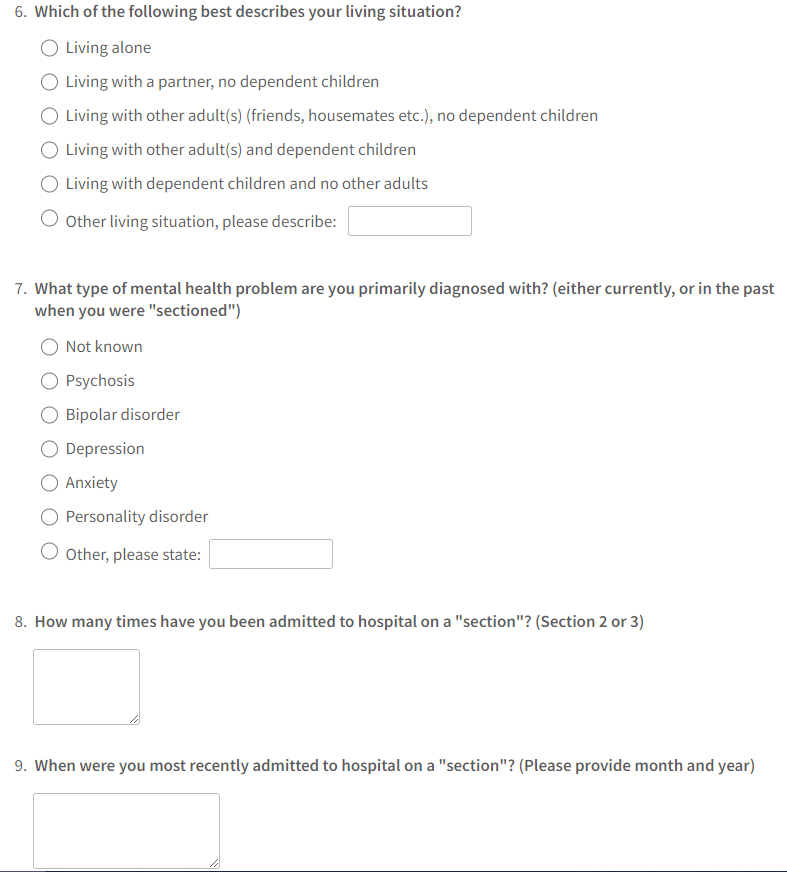

Supplement: S3 Appendix — (DOCX) [file pmen.0000417.s003.docx]
